# 

**Published:** 1849-02

**Authors:** 


### CONTENTS

OF NO. II.

---

### ORIGINAL COMMUNICATIONS.

ESSAYS AND CASES.

ART. I.—Lectures on Typhoid Fever, delivered in the Hotel Dieu Hospital of Lyons. By M. BRACHET. Translated by D. W. YANDELL, M. D., of Louisville, Ky.......93

ART. II.—A Case of Congenital Hydrocephalus. By WM. T. PRENTIS, M.D., of Lewisport, Kentucky..........105

ART. III.—A Case of Extra-Uterine Pregnancy. By Dr. W. W. HARBERT, of Shelby county, Kentucky..............110

ART. IV.—Chloroform in Dysmenorrhœa. By J. V. WITHERS, M.D., of Westpoint, Kentucky...................113

ART. V.—A Case of Amenorrhœa. By JAMES R. WILCOX, M.D., of Mile Grove, Indiana.........................116

ART. VI.—Belladonna as a Preventive of Scarlatina. By S. B. ROBINSON, M.D., of Rutherford county, Tenn. .......118

ART. VII.—A Case of Cholera in which Chloroform was successfully given internally. By R. S. STROTHER, M.D., of Bardstown, Kentucky. ............................119

ART. VIII.—Idiopathic Tetanus in a Horse successfully treated by Chloroform. By T. L. MADDIN, Student of Medicine in the University of Louisville................120

REVIEWS.

ART. IX.—Observations on the Pathology of Croup: with remarks on its Treatment by Topical Medications. By HORACE GREEN, A.M., M.D., etc., etc.................122

ART. X.—Report of the Standing Sanatory Committee of the Board of Health of the City of New York, on the subject of Asiatic Cholera, at present prevailing at the Quarantine Establishment of New York, at Staten Island ........ 133

ART. XI.—An Account of the most important Diseases peculiar to Women. By ROBERT GOOCHE, M. D., with Illustrations ........ 158

SELECTIONS FROM AMERICAN AND FOREIGN JOURNALS.

Membranous Croup ........ 159
Means of Applying Heat to Cholera Patients ........ 159
Progress of Cholera ........ 161
Statistics of Cholera ........ 161
The Cholera ........ 162
Epidemic Cholera ........ 166
Cholera in Granite Regions ........ 169
Chloroform in Cholera ........ 170
Influence of Russian Vapor-Baths on the Cholera ........ 170
Case of common continued (Typhoid) Fever.—Autopsy ........ 171
Diagnosis of Hypertrophy of the Brain from Chronic Hydrocephalus ........ 175
Prof. Mulder's Chemical Counterblast against the Potato as an article of Diet ........ 176

ORIGINAL INTELLIGENCE.

Cholera ........ 177
Where Cholera Prevails ........ 178
The Discoverer of Chloroform ........ 179
Mode of rendering Sulphate of Quinine tasteless ........ 179
A Second Medical School in Louisville ........ 180
Introductory Lectures ........ 180
Albany Medical School ........ 182
Outlines of a New System of Physiology ........ 182
Vegetation traced to Natural Causes ........ 183
Education of the Deaf and Dumb ........ 183
Indiana Lunatic Asylum ........ 183
American Medical Association ........ 184

